# Effect of Sprouting on the Phenolic Compounds, Glucosinolates, and Antioxidant Activity of Five *Camelina sativa* (L.) Crantz Cultivars

**DOI:** 10.3390/antiox12081495

**Published:** 2023-07-26

**Authors:** Elisabetta Bravi, Beatrice Falcinelli, Giorgia Mallia, Ombretta Marconi, Aritz Royo-Esnal, Paolo Benincasa

**Affiliations:** 1 Italian Brewing Research Centre, University of Perugia, 06126 Perugia, Italy; 2 Department of Agricultural, Food and Environmental Sciences, University of Perugia, 06125 Perugia, Italy; beatrice.falcinelli@unipg.it (B.F.); giorgia.mallia@unipg.it (G.M.); paolo.benincasa@unipg.it (P.B.); 3 Department of Agricultural and Forest Science and Engineering, University of Lleida, 25198 Lleida, Spain; aritz.royo@udl.cat

**Keywords:** *Camelina sativa* (L.) Crantz, seeds, sprouts, phenolic compounds, phenolic acids, glucosinolates, antioxidant capacity, UHPLC-UV, method validation

## Abstract

Sprouts are increasingly present in the human diet, being tasty and healthy foods high in antioxidant compounds. Although there is a body of literature on the sprouting of many plant species, *Camelina sativa* (L.) Crantz has not yet been studied for this purpose. This study aimed to characterize the main bioactive compounds and antioxidant potential of seeds and sprouts of five different Camelina cultivars (ALBA, CO46, CCE43, JOELLE, and VERA). In particular, the contents of phenolic compounds (PCs), phenolic acids (PAs), and glucosinolates (GLSs) were investigated. PCs, PAs, GLSs, and the antioxidant activity of seeds differed among cultivars and were greatly increased by sprouting. A PCA analysis underlined both the effect of the cultivar (PC2) and the germination (PC1) on the nutritional properties of Camelina. The best nutritional properties of seeds were observed for ALBA and CCE43, while the best nutritional properties of sprouts were recorded for CCE43 and JOELLE, since the latter cultivar showed a greater enhancement in phytochemical content and antioxidant activity with sprouting. Finally, a UHPLC-UV procedure for the analysis of GLSs in Camelina was developed and validated. The performance criteria of the proposed method demonstrated that it is useful for the analysis of GLSs in Camelina.

## 1. Introduction

Camelina (*Camelina sativa* (L.) Crantz) is an annual oilseed species with a short growth cycle (85–100 days when sown in spring), native to Eastern Europe and Western Asia and belonging to the *Brassicaceae* family. Camelina adapts well to various climatic and soil conditions, is highly resistant to diseases and pests, and can be cultivated as a cash crop with low input requirements for water, nutrients, and pesticides and a good stress tolerance [1,2,3,4]. Camelina seeds are rich in oil (30% to 49%), containing ω-3 and ω-6 fatty acids; in protein (24% to 31%); and in bioactive compounds, such as tocopherols, phytosterols, phenolic compounds (PCs), and glucosinolates (GLSs), which make them of interest for healthy human nutrition [5,6,7,8].

Despite the body of literature on the composition and nutritional properties of Camelina seeds, to the best of our knowledge, no information is available on the composition of Camelina sprouts. Sprouts, i.e., the young seedlings obtained from germination harvested a few days after sowing (DAS), are attracting increasingly more attention in the human diet because of the increased consumer interest in healthy, low-processed, and additive-free foods [9]. Given their peculiar characteristics such as a unique color, rich flavor, and appreciable content of bioactive substances, they could be used to enhance salads’ sensorial properties, to garnish a wide variety of high-quality products, or as supplements after drying or freeze-drying [10]. Thus, sprouting represents a simple way to boost the production of phytochemicals [11].

Vegetables belonging to the *Brassicaceae* family have received significant scientific consideration because of their health-promoting effects, such as reducing the risk for cardiovascular diseases and certain types of cancer, improving the immune system, and protecting against allergies [12]. In the Brassica species, PCs and GLSs are the main phytochemical classes [13]. In detail, PCs are substances that possess one or more aromatic rings with attached hydroxyl substituents, which categorize them into several subgroups (i.e., phenolic acids, flavonoids, etc.) [14]. The health benefits of phenolic compounds are well documented in the literature [15,16,17]. GLSs are plant-derived nitrogen- and sulfur-containing compounds classified as aliphatic, aromatic, or indole groups. Unlike PCs, GLSs are not all beneficial for human health; some can even work as antinutrients [16,18]. GLSs have been extensively studied due to their hydrolysis compounds (isothiocyanates, sulforaphane, and benzyl isothiocyanate) and indoles (indol-3-carbinol), which are associated with many health benefits. On the other hand, progoitrin is an “undesirable” glucosinolate because it is converted into goitrin (an antithyroid compound) after myrosinase hydrolysis [12,19]. For this reason, in recent years, the evaluation of biomass obtained from Brassica species has been based on the presence and abundance of any single GLS molecule rather than on the total GLS content.

Several scientific research works have reported that the content of bioactive compounds in *Brassicaceae* vegetables varies with the genotype, growth conditions, storage, and processing [20,21,22,23]. Biondi et al. underlined how the phytochemical load of broccoli depends upon both genetics and environment, and there is a large possibility of improving the quality of Brassica products through appropriate agronomic practices; nevertheless, the effects of the treatments are strictly genotype dependent [21]. As far as sprouts are concerned, Liu et al. reported that the PCs and antioxidant activity (DPPH and ABTS) values of 5-day-old broccoli sprouts were higher than those of the corresponding seeds [24].

Some researchers have also shown that, compared to other phytochemicals (e.g., PCs), GLSs behave in a way that is not always univocal during germination. Hanschen et al. reported a decreasing GLS content after germination, but also underlined that it can increase again. Moreover, GLS synthesis is strongly regulated by factors such as plant nutritional status and water availability [25]. Sarıkamıs et al. observed significant changes in the GLS content of cabbage and black radish during germination and decreases in GLSs upon imbibition were observed in both species [26]. In the same way, Gu et al. reported a decrease in germinating broccoli seeds, reaching the lowest value at 48 h, and a subsequent increase at 60 h [27]. Baenas et al. reported a general trend of decreasing over germination time for Brassica species [12]. The authors characterized nine varieties of *Brassicaceae* regarding their PC and GLS contents and in vitro antioxidant capacity and underlined the differences, both qualitative and quantitative, among genotypes. Moreover, the study highlighted how not all the GLSs of seeds are detected in sprouts and, on the other hand, some GLSs were present only in sprouts.

Moreover, Le et al. showed that PCs and GLSs are present in high amounts in seeds and during the first days of germination, exhibiting a 10-fold increase compared to commercial adult plants [28]. Pérez-Balibrea et al. studied the phytochemical content of different cultivars of broccoli, and their results showed significant differences in individual GLSs, PCs, and antioxidant capacity among the different cultivars. After germination, the authors underlined that sprout age and genotype should be considered as important features for broccoli sprouts for causing large differences in their phytochemical profile [29].

In *Brassica oleracea*, the type and concentration of individual GLSs appear to vary according to the variety, the plant parts in which they occurr, and the sprouting period of the seed. In particular, the content of alkyl GLSs decreases, whereas that of indol-3-ylmethylglucosinolates increases, throughout the sprouting period [30]. Perez-Balibrea et al. underlined that in *B. oleracea* var. *italica*, the GLS concentration was strongly influenced by germination, causing a rapid increase during the first three DAS and decreasing afterward [31]. Some authors have reported the GLS content of seeds, sprouts, and seedlings of white head cabbages and black radishes; one study demonstrated changes in GLSs from seed to sprout, where the content was maximum [26]. Vale et al. reported on variations in phenolic compound content in different Brassica vegetables; in the study, sprouting resulted in an overall increase in the PC content and antioxidant capacity, and although germination time was not a discriminating factor, higher germination times resulted in lower antioxidant capacity of the sprouts [32]. Falcinelli et al. investigated the effect of sprouting and increasing salinity on free and bound PCs, non-flavonoids, tannins, phenolic acids, and the antioxidant activity of rapeseed [33]. Sprouting and increasing the salinity slightly decreased all the studied fractions, while it markedly increased the free ones and the antioxidant activity. Thus, it is evident that, as for other plant species, in *Brassicaceae*, both the profile and the phytochemical content of seeds and sprouts are strongly affected by the genotype.

Despite that, there are many studies on the phytochemical composition of seeds and sprouts in the *Brassicaceae* family, to our knowledge, there are few studies on the chemical composition of Camelina seeds and none about the nutritional content of Camelina sprouts. On this basis, this study aimed to characterize seeds and sprouts of five Camelina cultivars for their PC and GLS contents and their antioxidant potentials.

## 2. Materials and Methods

### 2.1. Plant Material and Experimental Design

Seeds of five C. sativa cultivars (ALBA, CO46, CCE43, JOELLE, VERA) were used in this study. ALBA, VERA, and CCE43 were provided by Dr. Anìbal Capuano of the Camelina Company España, Madrid, Spain, while CO46 and JOELLE were provided by Dr. Russ W. Gesch of the United States Department of Agriculture (USDA-ARS) in Morris (MN, USA). Sprouting conditions were chosen in analogy with the method adopted by Benincasa et al. [34]. Briefly, seeds were incubated in plastic trays (1.5 g of seed per tray) containing filter paper laid over sterile cotton wetted with distilled water (75 mL) to guarantee constant water availability and prevent anoxia. The trays were closed with a perforated lid and placed in a growth chamber at 24 °C with a light/dark cycle (16/8 h) with a light intensity of 200 µmol photons m^−2^ s^−1^ according to a completely randomized block design with four replicates (trays). Sprouts were harvested at the stage of fully expanded cotyledons (6 DAS) by regrouping replicates two by two for chemical analyses. Fresh and dry weights and the lengths of shoots and roots of sprouts were measured for 10 individuals per replicate. The dry weight was measured following the Association of Official Analytical Chemists’ methods 925.10 [35]. The remaining sprout biomass of each replicate was kept separate and stored at −20 °C until chemical analysis. The germination percentage (G) and the mean germination time (MGT) were measured by running a separate germination test with three replicates (Petri dishes) of 100 seeds each per cultivar.

### 2.2. Chemicals

Methanol, water, and acetonitrile (HPLC grade), 2,2-diphenyl-1-picrylhydrazyl (DPPH), 2,2-azino-bis (3-ethylbenzothiazoline-6-sulfonic acid) diammonium salt (ABTS), 2,4,6-tris (2-pyridyl)-s-triazine (TPTZ), gallic, α-resorcylic, gentisic, p-hydroybenzoic, 2,6-dihydroxybenzoic, m-hydroxybenzoic, vanillic, salicylic, syringic acid, homovanillic, p-coumaric acid, m-coumaric, o-coumaric, ferulic, sinapic, caffeic, and chlorogenic acid, tyrosol, glucoarabin (9-methyl-sulfinyl-nonyl-glucosinolate; GS9), glucocamelinin (10-methyl-sulfinyl-decyl-glucosinolate; GS10) and homoglucocamelinin (11-methyl-sulfinyl-undecyl-glucosinolate; GS11) potassium salt, and tetrabutylammonium hydrogen sulfate (TBAHS) were purchased from Sigma-Aldrich (St. Louis, MO, USA). All other chemicals were of analytical grade.

### 2.3. Extraction of Free and Bound Forms of Phenolic Compounds and Glucosinolates

The entire extraction was carried out following the procedure of Bravi et al. [36]. Briefly, 1 g of the sample (ground seeds and frozen sprouts), for each replicate, was mixed with 5 mL of methanol, water, and acetic acid (70/29.5/0.5), homogenized, ultrasonicated, and centrifuged. The entire extraction was repeated twice, the supernatants collected, evaporated to dryness, and dissolved in 1 mL of 30% methanol in eluent A (0.1 M citric acid and 0.2 M sodium hydrogen phosphate; 85:15; *v*/*v*) before the determination of the free total PCs, free phenolic acids (PAs), GLSs, and antioxidant activities (DPPH, FRAP, and ABTS). The bound fraction was obtained by alkaline hydrolysis (4 M sodium hydroxide, sonicating for 40 min, and left overnight at room temperature) of the solid residues left after extraction of free phenolic forms and GLSs. After the alkaline hydrolysis, the hydrolyzed mixture was adjusted to pH 2 and extracted with ethyl acetate. The supernatants were collected, evaporated under a vacuum, and dissolved in 1 mL of 30% methanol in eluent A. The bound fractions were used to determine the content of bound PCs, bound PAs, and antioxidant activity of bound fractions.

### 2.4. Total Phenolic Compounds

The total PC (TPCs) content of free and bound fractions extracted from *Camelina* seeds and sprouts was determined following the Folin–Ciocalteu method [37]. An aliquot (0.4 mL) of free and bound phenolic extracts was added to 2 mL of Folin–Ciocalteu reagent (1:10, *v*:*v*) and 1.6 mL of sodium carbonate (7.5%) and incubated in the dark for two hours. The absorbance was measured at 765 nm against a blank sample. Gallic acid (GA) was used as the standard to calibrate the method. The results were expressed as mg of GA equivalent (GAE) per g of sample dry matter (mg GAE g^−1^ dm). Total PCs levels were calculated as the sum of the free and bound fractions.

### 2.5. Determination of Phenolic Acids

PAs were analyzed following the method developed by Bravi et al. using a UHPLC system consisting of a Knauer 3950 sampler with a 10 μL loop, an Azura P 6.1 L quaternary pump (Knauer, Berlin, Germany) coupled with an eight-channel Azura MWD 2.1 L UV-Vis detector [36]. Separation was performed at 25 °C and a flow rate of 0.4 mL min^−1^ using a SunShell C18 column (ChromaNik Technologies Inc., Osaka, Japan, 50 mm × 2.1 mm ID). Mobile phase A was 0.1 M citric acid and 0.2 M sodium hydrogen phosphate (85/15; *v*/*v*) at pH 2.88 and mobile phase B was phase A, methanol, and acetonitrile (30/20/50, *v*/*v*/*v*) at pH 3.44. The elution gradient was the following: phase A 90% (0 min), 100% (2 min), 70% (8 min), 50% (10 min), 20% (12 min), and 90% (12.5 min). The wavelengths were 254, 278, and 324 nm. A stock solution of 100 ug/mL of a mixture of the phenolic acids considered in methanol/eluent A (30:70, *v*:*v*) was used to prepare the working solutions for the external standard calibration. The calibration curves were constructed with linearity between 0.5 and 5 μg/mL. The calibration curve of each phenolic compound was plotted at the maximum UV absorption for a given substance (270 nm for 3,4,5-trihydroxybenzoic, homovanillic, syringic, m-coumaric, and o-coumaric; 254 nm for 3,5-dihydroxybenzoic, 4-hydroxybenzoic, 2,6-dihydroxybenzoic, 3-hydroxybenzoic, and vanillic; 324 nm for 2,5-dihydroxybenzoic, caffeic, chlorogenic, p-coumaric, salicylic, ferulic, and sinapic).

### 2.6. Antioxidant Activity

The antioxidant activity of seeds and sprouts of the five cultivars of Camelina was measured, according to Benincasa et al., using ABTS, DPPH, and FRAP assays and Trolox as standard for the calibration curves [38]. Briefly, the ABTS involves the addition of an ABTS solution to an aliquot of the free and bound phenolic extract. After 2 h in the dark, the absorbance was read at 734 nm. For the DPPH assay, DPPH solution was added to an aliquot of the free or bound phenolic extract and the absorbance was read after 30 min at 515 nm. Finally, for the FRAP assay, an aliquot of the phenolic extract was mixed with the FRAP working solution and warmed at 37 °C in the dark for 30 min. The absorbance was read at 593 nm.

The results of the three assays were expressed as Trolox equivalents (TE) g^−1^ on the dry matter (dm) of the sample. The antioxidant activity of each cultivar was measured for the free and bound fractions of seeds and sprouts, while the total antioxidant activity was calculated as the sum of the activities of the free and the bound fraction. Moreover, the concentration of the antioxidant-containing substance required to scavenge 50% of the initial redox/radical systems used for antioxidant evaluation, namely IC50, was calculated for each antioxidant assay. The lower the IC50 value, the higher the antioxidant activity of the substance [36].

### 2.7. Glucosinolates

GLSs were analyzed using the same UHPLC system used for the determination of PAs. The separation was performed using a Luna Omega PS-C18 column (Phenomenex Inc., Torrance, CA, USA, 50 mm × 2.1 mm ID) at 40 °C and a flow rate of 0.4 mL min^−1^. Phase A was 0.005 M TBAHS and phase B was methanol. Chromatographic separation was achieved using the following elution gradient: mobile phase A 70% (0 min), 60% (3 min), 60% (5 min), 70% (6 min), and 70% (8 min). The wavelengths of the four channels used for detection were 210, 220, 225, 237, and 280 nm. Clarity Chromatography software (8.6 version) for Windows (DataApex, Prague, Czech Republic) was used for data acquisition and elaboration. The external standard method was used for the calibration and a 100 µg mL^−1^ stock solution of a mixture of the GLSs in methanol/phase A (30:70, *v*:*v*) was used to prepare the working solutions. Calibration curves were constructed for each standard compound, with a linearity between 1 and 20 μg mL^−1^.

### 2.8. UHPLC Method Validation

The validation of the UHPLC-UV method of the quantitative determination of GLSs in *Camelina sativa* L. was carried out to evaluate its performance, to confirm that the method used meets the requirements, and to ensure the reliability of the results [39]. The following validation parameters were considered: (i) system suitability; (ii) linearity; (iii) homoscedasticity; (iv) limit of detection (LOD) and limit of quantification (LOQ); (v) accuracy (trueness and precision); and (vi) uncertainty.

The system suitability was calculated by ten injections of the same standard solution containing GLSs. The parameters calculated were the relative standard deviations (% RSD) of retention time (tR) and peak area (A) for each GLS, the number of theoretical plates (N), and the tailing factor (T). The linearity was tested in the GLS concentration range of 1–20 µg mL^−1^. According to the Eurachem Guide, the solutions were injected ten times, external calibration curves for each GLS were constructed at seven calibration levels, and the correlation coefficient (R) for each plot was calculated [40]. The Cochran test was used to evaluate the homoscedasticity of the calibration plots. The test statistic calculated is the ratio of the major variance to the sum of all variances (Cc, C computed) [36]. The limit of detection (LOD, three times the signal-to-noise ratio) and the limit of quantification (LOQ, ten times the signal-to-noise ratio) of each GLS were also calculated [41]. To study the accuracy of the results, the trueness and the precision were calculated. The trueness, which means how close the average of ten replicates is to a reference value, was expressed as the relative peak recovery, R′% [42]. Precision (how close the results are to each other, i.e., how replicable they are) was expressed by the statistical parameter relative standard deviation percentage, RSD%, which is a measure of the spread of the results. Therefore, relative standard deviations of repeatability (% RSDr) were calculated. Finally, the uncertainty was tested by calculating the Horwitz ratio (HorRat) value, a parameter proper for an inter-laboratory validation study [41].

### 2.9. Statistical Analysis

Statistical analysis was performed by a two-way ANOVA using R software 4.3.0 [43]. Values were expressed as mean ± standard error of two biological replicates, each replicate represented the mean of three analytical replicates. The significance of the effect of the variety (CV), the growth stage (*GS*), their interaction (CV × *GS*), and the *LSD* of their interaction were reported. The means were compared by means of the minimum significant Fisher difference of the interaction (*LSD*) at *p* ≤ 0.05.

A principal component analysis (PCA) was performed using the PLS-Toolbox (Eigenvector Research, Inc., B. Wise and N. Gallagher, Manson, WA, USA) with MATLAB v. 7.6.0. (The Mathworks Inc., Natick, MA, USA). The data were mean-centered before the PCA analysis in order to correct the different unit variability among the measure variables.

## 3. Results and Discussions

### 3.1. Germination and Sprout Growth

Table 1 reports the results for the germination and sprout growth parameters of the five Camelina cultivars. The total germination percentage ranged between 88% (ALBA) and 98% (CCE43 and JOELLE). The mean germination time (MGT) ranged between 1.1 and 2.0 days. Therefore, the germination performance of Camelina appears in line with that of rapeseed—even faster—indicating it is suitable for sprouting. Shoot lengths and especially root lengths varied among cultivars, as well as the fresh weight of individual sprouts (from 9.6 mg in VERA to 14.8 mg in CO46). Overall, Camelina sprouts were much smaller than the values of those of rapeseed obtained by Falcinelli et al., roughly half in weight for a similar growth stage and sprouting conditions (6 DAS for Camelina, 7 DAS for late sprouts of rapeseed obtained with distilled water) [33].

### 3.2. Phytochemical Content and Antioxidant Activity

#### 3.2.1. Total Phenolic Compounds

Interesting concentrations of PCs were found in both Camelina seeds and sprouts. The contents (on a dry matter, dm) of PCs in Camelina seeds and sprouts are reported in Table 2. The values ranged between 9.5 and 11.4 mg GAE g^−1^ for seeds and between 14.8 and 21.5 mg GAE g^−1^ for sprouts. In both seeds and sprouts, free PCs (FPCs) represented the main fraction of total PCs (TPCs), on average 80% of the total fraction in seeds and 92% in sprouts. In seeds, FPCs varied between 7.5 (VERA) and 9.2 mg GAE g^−1^ (ALBA), while bound PCs (BPCs) ranged between 1.3 (CO46) and 3.4 mg GAE g^−1^ (CCE43). To our knowledge, few studies are reported in the literature on Camelina seed composition, and nothing is reported for the sprouts. Terpinc et al. reported similar content of PCs in Camelina seeds [44]. Quezada and Cherian found slightly lower contents of TPCs in Camelina seeds, the values were 1 mg GAE g^−1^ by ethyl acetate extraction and 3 mg GAE g^−1^ by methanolic extraction [45]. Karamać et al., studied the amount of PCs in the *Camelina* plant at different growth stages; they found concentrations ranging between 1.4 and 3.1 mg GAE g^−1^ (on a fresh weight basis) [46]. The differences in the obtained results are probably due to the different solvents used for the extraction and to the different genotypes of Camelina analyzed in the different research studies. As expected, passing from seeds to sprouts, the amount of PCs increased significantly in all the cultivars. In particular, the increase involved the FPCs, while BPCs did not change or slightly decrease (i.e., in ALBA; CCE43; VERA). Therefore, the TPC fraction in sprouts was significantly higher compared to seeds, and the amount of the increase varied between cultivars, i.e., the lowest one was +23% for ALBA while the highest one was +55% for JOELLE.

#### 3.2.2. Phenolic Acids

The total PA (TPA) content in the seeds of Camelina ranged between 496 (CO46) and 1274 (ALBA) µg g^−1^ dm, accounting for the main fraction (58–84%) of free PAs (FPAs); the results are reported in Table 2. The main representative FPAs in seeds were homovanillic, sinapic, ferulic, and syringic acid, in decreasing order of concentration. The PAs of the bound fraction (BPAs) were, in order of decreasing concentration, sinapic, 3.5-dihydroxybenzoic, salicylic, ferulic, and p-coumaric acid. With sprouting, the amount and the types of PAs increased (homovanillic acid disappeared after sprouting in all cultivars, and ferulic acid disappeared in JOELLE). In particular, the TPA content of sprouts ranged from 2206 (JOELLE) to 3959 (CCE43) µg g^−1^ dm. The extent of the increase varied between cultivars, i.e., the lowest one was +16% for CCE43 while the highest was +46% for ALBA. Even in sprouts, as well as in seeds, the main fraction of TPAs was the free fraction (55–77%). The main representative FPAs were chlorogenic, salicylic, syringic, sinapic, p-coumaric, 3-hydroxybenzoic, vanillic, and ferulic acid, in decreasing order of concentration. BPAs also included 3.5-dihydroxybenzoic, m-coumaric, and caffeic acid (Table 2). All the PAs found in Camelina seeds and sprouts have beneficial effects on human health, they act as antioxidants and anti-inflammatory, antimicrobial, antitumor, antiviral, antifungal, antidiabetic, and hepatoprotective agents [47,48,49]. The germination improved the health properties of Camelina sativa by increasing the content and the number of types of PAs.

#### 3.2.3. Glucosinolates

GLSs represent a class of compounds mainly found in the order of *Brassicales*. All GLSs are made up of three different parts: β-thioglucose, thiohydroximate-O-sulfonate, and a variable aglycone side chain derived from an α-amino acid, which classifies the molecule as either aliphatic, indole, or aromatic. GLSs are also classified on the basis of the amino acid precursors, e.g., Trp-, Ile-, Met-, Leu-, Val-, Glu-, Phe-, or Ala-derived [50]. The aglycone side chain confers different biological functions to GLSs. Several epidemiological studies attest that increasing GLS intake from cruciferous vegetables moderates the possibility of developing several diseases, in particular cancer [51,52].

There were three GLS molecules found in the different cultivars of Camelina: GS9, GS10, and GS11. The average results (dm) and the standard errors for the GLS content of Camelina seeds and sprouts are shown in Table 3. The content of total GLSs ranged between 2.34 (CCE43) and 6.16 mg g^−1^ (ALBA). The most represented GLSs were GS9 and GS10 (on average among cultivars, at 45% and 42%, respectively, of the total GLS content). Sprouting caused a significant increase in the total GLS content in all cultivars, except for VERA where the variation was negligible. The increase varied from +8% (ALBA) to +39% (CCE43). However, the increase concerned only GS9, whereas GS10 decreased in all cultivars. As compared to seeds, GS9 increased 2- and 3-fold in VERA and in CCE43, respectively, while the content of GS10 strongly decreased (from −32% in JOELLE to −91% in ALBA). It is possible that *Camelina* synthetizes GS9 during germination as a consequence of the reactivation of its metabolism and the restoration of embryo growth. The GS11 content, already very low in seeds, decreased further with sprouting. Thus, in sprouts, GS9 became the most prevalent GLS compound, representing 87% of the total GLS content (on average over cultivars).

The results of GLS obtained in the present study are in line with the literature [53]. Some authors found similar GLS contents in seeds of *Camelina sativa* (L.) Crantz [54]. The authors highlighted that the main GLS in the genotypes analyzed was GS10, and the total GLS content in the available collection varied from 13.2 to 36.2 mmol g^−1^ dry seed. The authors noted an evident influence of the environment on GLS content. Moreover, Berhow et al. investigated the content of GLSs in Camelina seeds, and they highlighted levels of GS9, GS10, and GS11 similar to those observed in our Camelina seeds [55]. Moreover, the authors also examined the levels of GLSs in Camelina during the course of sprouting and, contrarily to our findings, they observed a general decrease in the levels of total GLSs; at the end of the trial, their sprouts did not contain measurable levels of GS9 and GS10. In addition, the concentration of GS9 increased and GS10 underwent a significant decrease during sprouting, even in an experiment of Berhow et al. [55].

GLSs found in Camelina seeds and sprouts investigated in this research were all methionine-derived compounds, which are the most commonly consumed within human diets. These compounds are generally known for their role in interfering with key processes of carcinogenesis and as an active part in the protection of cells from redox damage that leads to the development of chronic inflammatory diseases [51,56,57,58]. Moreover, these typologies of GLSs (Met-derived) are the ones that confer the typical, distinctive flavor through the development of their breakdown products [59].

#### 3.2.4. Antioxidant Activity

**Figure 1:**
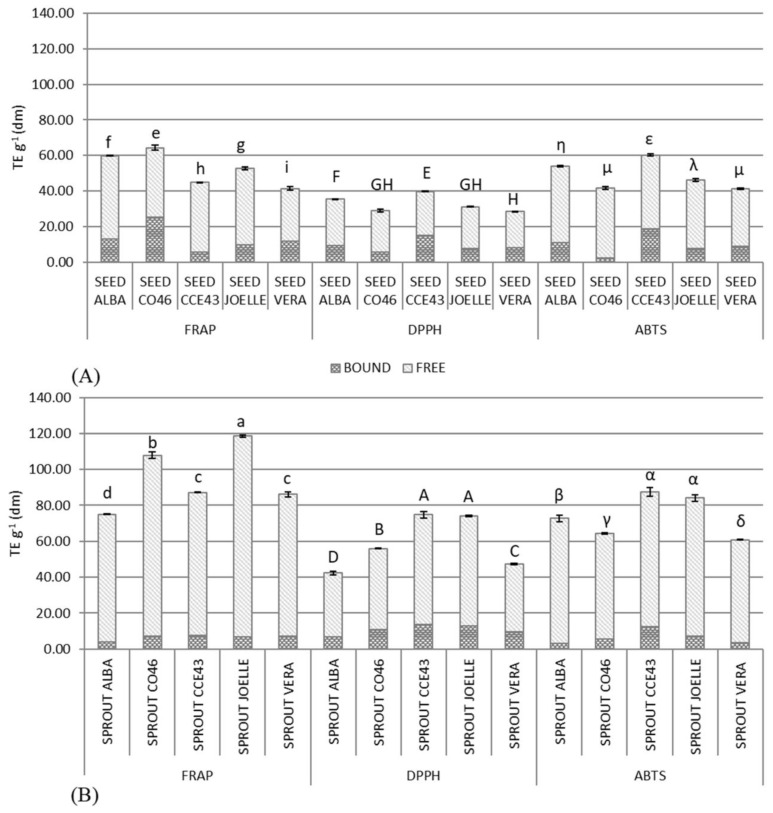
Antioxidant activities (TE g^−1^ dm) as the sum of the bound (dark grey) and free (light grey) fraction in seed (**A**) and sprout (**B**) samples of the five cultivars (ALBA, CO46, CCE43, JOELLE, and VERA) of *Camelina sativa* (L.) Crantz. n = 2; TE = Trolox equivalent; dm = dry matter; different letters within lowercase, uppercase, and Greek letters indicate significant (*p* = 0.05) differences between samples (including both seeds and sprouts) within each test (FRAP, DPPH, and ABTS).

Figure 1 reports the results of three antioxidant assays (FRAP, DPPH, and ABTS) of Camelina seeds and sprouts. Two or more assays are usually considered necessary to avoid any influences of the redox or radical system involved in the mechanism of each single antioxidant test. The results showed that each cultivar’s seeds and sprouts have an interesting antioxidant power. As known, the peculiar molecules that characterize seeds and sprouts of the *Brassicaceae* family also confer antioxidant activity [60,61]. The antioxidant activity of free, bound, and total fractions, measured by DPPH, FRAP, and ABTS, varied among cultivars and growth stages.

In all assays, the antioxidant activity of the free fraction for both seeds and sprouts of all cultivars was higher than that of the bound one. This agrees with the fact that FCPs and FPAs were the most represented compared to the bound fractions and that GLSs are also represented in the free fraction.

Moreover, as expected, the antioxidant activity of the free and total phenolic fraction increased passing from seeds to sprouts in all cultivars. The activity of the bound fraction had different trends among assays. DPPH values increased (except for ALBA and CCE43), while FRAP and ABTS values decreased (except for ABTS in CO46). Overall, based on the three assays, the highest levels of antioxidant activities in seeds were measured for ALBA and CCE43. The results are positively correlated with the content of TPCs, PAs, and GLSs.

Concerning sprouts, based on the three assays, the highest antioxidant activity was recorded in JOELLE, CCE43, and CO46. These results are positively correlated with TPCs and GLSs. Therefore, the ranking of cultivars for antioxidant activity in seeds does not necessarily reflect the ranking for sprouts. This evidence was also reported in previous studies on the sprouting of other plant species, for example, in pomegranate [62] and citrus species [63].

**Figure 2:**
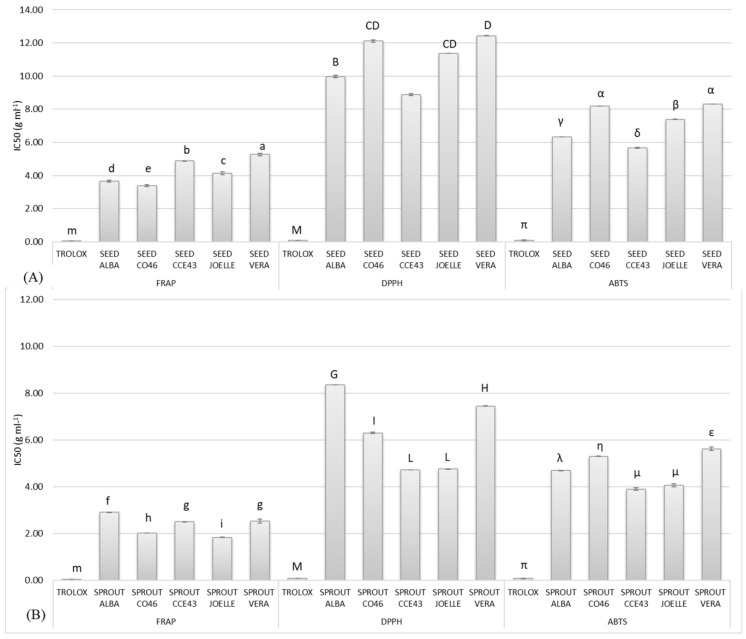
Median inhibitory concentration (IC50) (g mL^−1^) of seed (**A**) and sprout (**B**) samples of the five cultivars (ALBA, CO46, CCE43, JOELLE, and VERA) of *Camelina sativa* (L.) Crantz, n = 2. Different letters within lowercase, uppercase, and Greek letters indicate significant (*p* = 0.05) differences between samples (including both seeds and sprouts) within each test (FRAP, DPPH, and ABTS).

The IC50, that is the amount of Camelina seeds and sprouts necessary to inhibit up to 50% of the probe in each selected antioxidant assay, was also measured [36]. The results are reported in Figure 2. The IC50 values for seeds ranged between 3.40 and 5.27 g mL^−1^ for FRAP, 8.88 and 12.43 g mL^−1^ for DPPH, and 5.67 and 8.31 g mL^−1^ for ABTS. The IC50 values for sprouts were at least 1.2-fold higher in comparison with seeds, varying between 1.84 and 2.91 g mL^−1^ for FRAP, 4.72 and 8.36 g mL^−1^ for DPPH, and 3.91 and 5.63 g mL^−1^ for ABTS. As discussed before, these results are likely related to the higher content of TPCs, PAs, and GLSs of sprouts as compared to seeds.

Few studies are found in the literature on the antioxidant activity of Camelina seeds, and none on sprouts. Rahman et al. reported a reducing power (RP) of 105 TE g^−1^ of the defatted sample [64]. The RP was measured with a method slightly different to our FRAP assay. The slight differences in the results can be explained by considering the differences in the method and the different genotypes of *Camelina* studied. Mieriņa et al. investigated Camelina oil and reported a DPPH value similar to that obtained in our study, but the matrix analyzed was different [65].

#### 3.2.5. PCA Analysis

**Figure 3:**
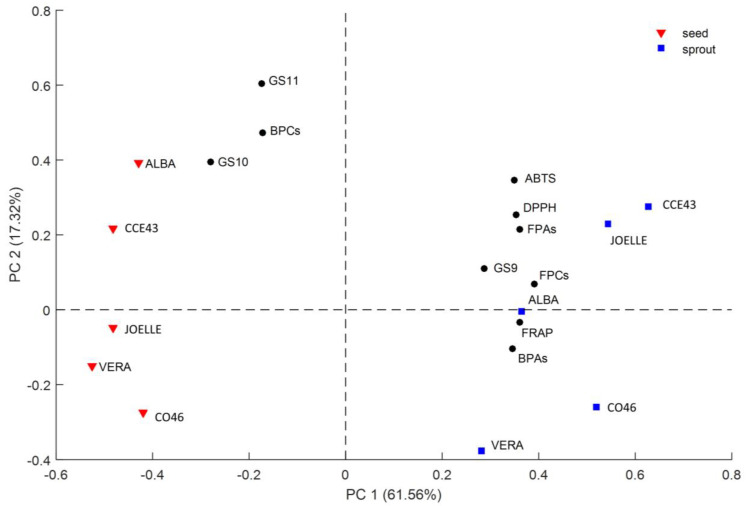
The score and loading biplot of the first and the second principal components (PC1 and PC2) of PCA on the 10 samples (five varieties for two different growth stages: seed and sprout). FPAs = free phenolic acids, BPAs = bound phenolic acids, FPCs = free phenolic compounds, BPCs = bound phenolic compounds, GS9 = glucoarabin, GS10 = glucocamelinin, GS11 = homoglucocamelinin.

A PCA was performed on a 10 × 10 matrix from the analyzed parameters (FPCs, BPCs, FPAs, BPAs, GS9, GS10, GS11, FRAP, DPPH, and ABTS values) for all the 10 samples (five cultivars for both seed and sprout stage). The first two principal components explained 78.88% of the total variation. The score and loading biplot of the first and the second principal components (PC1 and PC2) are shown in Figure 3. Concerning the scores, which represent the samples, PC1 discriminates between seeds on the left and sprouts on the right. Observing the loadings, representing the analyzed parameters, the plot shows that seeds are mainly characterized by BPCs, GS10, and GS11, which decrease during germination. On the other hand, the sprouts are mainly described by high values of FPCs, FPAs, BPAs, GS9, and antioxidant capacity, which increase during germination.

PC2 discriminates samples on the basis of cultivar, and the analyzed parameters which mostly influence this trend are BPCs, GS10, and GS11, but also FPAs, DPPH, and ABTS.

The seeds of ALBA and CCE43 are in the fourth quadrant for positive values of PC2 because they are characterized by high values of DPPH and ABTS, BPCs (CCE43), FPAs, and GLSs (ALBA). The CCE43 sprouts are in the first quadrant and are characterized by the highest TPAs, DPPH, and ABTS values, which increased during germination. The ALBA sprouts are in the lower part of the first quadrant, and they show low values of all the analyzed parameters when compared with the other sprouts, except for GS9, whose value was already high in seeds.

The seeds of JOELLE are in the upper part of the third quadrant, and they are characterized by mean values of TPCs, GLSs, and antioxidant capacity; they also show the lowest value of FPAs and the highest value of BPAs. The corresponding sprouts are in the first quadrant because they are characterized by a high antioxidant capacity, which significantly increased during germination; it also shows high values of GS9 and FPCs.

The seeds of CO46 are in the bottom part of the third quadrant because they are characterized by low values of all the considered parameters with the exception of FRAP, whose high value is likely due to bioactive compounds other than those analyzed in this study. The corresponding sprouts are in the bottom part of the second quadrant because they show a trend similar to that of seeds.

A similar trend is also followed by VERA, whose seeds and sprouts are in the bottom part of the plot because they are characterized by low values for all the analyzed parameters.

#### 3.2.6. Method Validation

Table 4 reports the results of the validation of the GLS method used in this study for analyzing GLS content in seeds and sprouts. The % RSD values of the retention time and peak area for each GLS were calculated to assess the system suitability, together with the number of theoretical plates (N) and the tailing factor (T). The % RSD of retention time ranged between 0.2 and 0.6%, and the % RSD of peak area between 1.3 and 1.4%. The % RSD of N and T was from 1.0 and 1.4%. The results for the suitability test were in the range of the acceptance criteria (approximately 1%), confirming the suitability of the system employed [36]. The quantification of GLSs was carried out using an external standard calibration method and the linearity was verified through the calculation of the R values between 0.9980 and 0.9984 and the relative intercepts that were very close to zero. The homoscedasticity of the calibration plots was verified by Cochran’s test. The calculated values (Cc) were lower than the C values tabulated (Ct), as listed in Cochran’s table for level of significance of α = 0.01, confirming the homoscedasticity of each plot (Table 4). Finally, the LOD ranged between 0.025 and 0.052 µg mL^−1^, and the LOQ was between 0.083 and 0.174 µg mL^−1^. Furthermore, to assess the efficiency of the whole procedure, the Camelina sprouts were spiked with different levels of GLSs and analyzed. The entire analysis, extraction, and UHPLC-UV analysis was replicated ten times (n = 10). The relative spike recovery R′% was calculated by analyzing spiked and unspiked samples ten times for three concentration levels (1, 5, and 15 mg g^−1^ of the three considered GLSs), and the obtained results allowed to verify the trueness of the method. The trueness of the method ranged between 92 and 102% (Table 5). The trueness values were in the right range according to AOAC guidelines considering that the content of GLSs in Camelina sprouts reaches the level of mg g^−1^. The expected recovery limit for this range of concentrations is between 90% and 108% [66]. The precision of the UHPLC method was evaluated by calculating the relative standard deviation of repeatability (%RSDr) and, as shown in Table 5, the calculated values were within the acceptability criteria range (±2%) [67]. Finally, the uncertainty was evaluated by calculating the Horwitz ratio value (HorRat); the accepted values for repeatability conditions for within-laboratory relative standard deviations are between 0.3 and 1.3. The obtained results, reported in Table 5, ranged between 0.2 and 0.5; consistent deviation from the ratio on the low side (values < 0.3 or 0.5) may indicate excellence experience and training, there are no significant differences between the coefficients of variation, so variances are homogenous (AOAC, 2013).

## 4. Conclusions

This study confirms the valuable composition of Camelina seeds, demonstrating their high contents of PCs, PAs, and GLSs and high antioxidant activities in all five tested cultivars. The free fraction was the most represented in both phenolic compounds (on average 80%) and phenolic acids (on average 74%). The GLSs found in seeds were GS9, GS10, and GS11, with the former two as the most represented (45% and 42% on average over cultivars). The content of bioactive compounds in seeds was significantly influenced by the cultivar of Camelina. Moreover, this study highlights that sprouting, which involves oxidative stress for the plant and the consequent synthesis of high amounts of antioxidant compounds, caused an increase in the content of PCs, including PAs, and in particular their free fraction (92% of total PCs and 72% of total PAs, respectively) and GLSs (in particular GS9). The extent of this increase during sprouting was significantly influenced by the cultivar. Since the greatest part of GLSs detected in sprouts was represented solely by GS9 (87% of the total, on average over cultivars), it can be deduced that Camelina synthetizes GS9 during germination. Overall, the GLSs found in the Camelina cultivars investigated in this research were all methionine-derived compounds, which are generally known for their anti-carcinogenic role and as protectors from the development of chronic inflammatory diseases. Moreover, these typologies of GLSs are related to the typical and distinctive “bite” flavor. Both seeds and sprouts of all cultivars showed an interesting antioxidant power. The antioxidant activity of free, bound, and total phenolic fractions, measured by DPPH, FRAP, and ABTS, varied among cultivars and increased passing from seeds to sprouts. For both seeds and sprouts, the antioxidant activity of the free fraction was higher than that of the bound one.

The findings of this study (i) demonstrate that Camelina is a plant species suitable for sprouting purposes; (ii) confirm the effectiveness of sprouting in increasing the content of bioactive compounds and the nutritional value of Camelina; and iii) highlight the differences due to genotype in terms of the germination performance and nutritional characteristics of Camelina sprouts.

The PCA analysis of the results clearly shows that the best phytochemical content and antioxidant activity in seeds was found in ALBA and CCE43, while the best phytochemical composition in sprouts was found in CCE43 and JOELLE, indicating that sprouting may enhance the composition of PCs and GLSs and the antioxidant activity differently depending on the cultivar.

Finally, this work allowed the development of a simple and reliable UHPLC-UV method for the quantitative determination of GLSs. A Cochran’s test confirmed the homoscedasticity of the calibration plots. The trueness, precision, and uncertainty results were within the acceptability criteria range (92 to 102%, 1.4 to 2.4%, and 0.2 to 0.5, respectively). Overall, the performance criteria of the proposed method demonstrated that the method is suitable for the analysis of GLSs in Camelina seeds and sprouts.

## Figures and Tables

**Table 1:** Total germination% (G), mean germination time (MGT) expressed as days after sowing (DAS), individual shoot and root length, fresh weight, and moisture content (%) of five *Camelina sativa* (L.) Crantz cultivars (ALBA, CO46, CCE43, JOELLE, and VERA).

Cultivar	G (%)	MGT (Days)	Shoot Length (mm)	Root Length (mm)	Individual Sprout Fresh Weight (mg)	Moisture (%)
ALBA	88 ± 0.9	1.9 ± 0.05	6.0 ± 0.23	16.2 ± 0.69	9.9 ± 0.09	85.2 ± 1.10
CO46	96 ± 0.9	2.0 ± 0.04	6.13 ± 0.23	38.5 ± 0.65	14.8 ± 0.81	91.3 ± 1.29
CCE43	98 ± 0.9	1.1 ± 0.04	7.84 ± 0.61	24.4 ± 1.33	11.6 ± 0.49	93.2 ± 0.41
JOELLE	98 ± 0.3	2.1 ± 0.04	7.26 ± 0.31	22.6 ± 0.49	11.0 ± 0.84	90.3 ± 1.27
VERA	93 ± 0.6	1.3 ± 0.03	7.32 ± 0.22	32.1 ± 1.05	9.6 ± 0.43	90.7 ± 1.44
Significance of ANOVA	-	**	*	**	**	-
*LSD*_0.05_	-	0.124	1.272	3.249	2.171	-

Means of n = 3 replicates ± standard errors are depicted for G% and MGT, while means of n = 2 replicates ± standard errors are depicted for sprout growth data; ** significant at *p* ≤ 0.01; * significant at *p* ≤ 0.05.

**Table 2:** Contents of free (FPC), bound (BPC), and total (TPC) polyphenols (mg GAE g^−1^ dm) and profile of free (FPA), bound (BPA), and total (TPA) phenolic acids (µg g^−1^ dm) in seeds and sprouts of five *Camelina sativa* (L.) Crantz cultivars (ALBA, CO46, CCE43, JOELLE, and VERA). Means of n = 2 replicates ± standard errors are depicted. *G* = genotype; *GS* = growing stage; *LSD*: least significant difference of *G* × *GS* interaction. Values followed by different lowercase letters are significantly different for *p* = 0.05. Significance of ANOVA: ** significant at *p* ≤ 0.01; * significant at *p* ≤ 0.05.

	Seeds					Sprouts					Significance			
Phenolic Compounds	ALBA	CO46	CCE43	JOELLE	VERA	ALBA	CO46	CCE43	JOELLE	VERA	*G*	*GS*	*G × GS*	*LSD*_0.05_
FPC (mg GAEg^−1^ dm)	9.23 ± 0.16 ^d^	8.39 ± 0.293 ^de^	7.95 ± 0.23 ^ef^	7.95 ± 0.46 ^ef^	7.45 ± 0.05 ^f^	13.76 ± 0.14 ^c^	18.87 ± 0.15 ^b^	18.58 ± 0.38 ^b^	19.87 ± 0.15 ^a^	13.67 ± 0.48 ^c^	**	**	**	0.900
BPC	2.18 ± 0.22 ^bc^	1.29 ± 0.16 ^efg^	3.36 ± 0.15 ^a^	1.71 ± 0.16 ^cde^	2.06 ± 0.27 ^bcd^	1.05 ± 0.07 ^g^	1.20 ± 0.05 ^fg^	2.23 ± 0.24 ^b^	1.66 ± 0.08 ^def^	1.29 ± 0.05 ^efg^	**	**	*	0.512
TPC	11.41 ± 0.46 ^d^	9.69 ± 0.60 ^e^	11.31 ± 0.50 ^d^	9.67 ± 0.85 ^e^	9.51 ± 0.35 ^e^	14.81 ± 0.24 ^c^	20.06 ± 0.12 ^b^	20.81 ± 0.32 ^ab^	21.53 ± 0.16 ^ab^	14.96 ± 0.19 ^c^	**	**	**	1.138
FPA (µg g^−1^ dm)														
Homovanillic	1116.31 ± 1.30 ^a^	330.95 ± 3.77 ^d^	471.49 ± 3.2.8 ^c^	279.31 ± 1.17 ^e^	544.86 ± 2.66 ^b^	-	-	-	-	-	**	**	**	5.90
3- hydroxybenzoic	-	-	-	-	-	48.66 ± 0.16 ^b^	-	143.65 ± 0.33 ^a^	-	-	**	**	**	0.36
Vanillic	-	-	-	-	-	24.63 ± 0.42 ^d^	27.37 ± 0.96 ^c^	61.10 ± 1.11 ^a^	-	42.54 ± 0.18 ^b^	**	**	**	1.53
Syringic	2.07 ± 0.02 ^f^	2.27 ± 0.03 ^f^	1.12 ± 0.03 ^f^	3.30 ± 0.13 ^f^	2.01 ± 0.01 ^f^	291.47 ± 2.20 ^c^	530.75 ± 0.61 ^a^	178.43 ± 1.21 ^e^	210.67 ± 4.08 ^c^	466.71 ± 3.62 ^b^	**	**	**	6.01
Salicylic	-	-	-	-	-	292.22 ± 11.45 ^b^	258.08 ± 7.92 ^c^	291.89 ± 6.52 ^b^	365.60 ± 0.85 ^a^	195.51 ± 3.03 ^d^	**	**	**	21.91
Chlorogenic	-	-	-	-	-	1275.62 ± 2.21 ^b^	1059.88 ± 9.56 ^c^	2161.59 ± 17.61 ^a^	945.09 ± 1.06 ^d^	423.87 ± 8.72 ^e^	**	**	**	3.83
p-coumaric	-	-	-	-	-	65.54 ± 1.32 ^b^	52.27 ± 1.57 ^c^	30.87 ± 2.01 ^d^	80.92 ± 0.78 ^a^	31.17 ± 2.43 ^d^	**	**	**	15.64
Ferulic	2.75 ± 0.10 ^d^	3.48 ± 0.09 ^d^	1.95 ± 0.15 ^d^	1.89 ± 0.22 ^de^	2.63 ± 0.02 ^d^	23.87 ± 1.09 ^b^	21.99 ± 0.36 ^b^	26.40 ± 1.44 ^a^	-	18.43 ± 0.73 ^c^	**	**	**	2.00
Sinapic	46.49 ± 0.24 ^de^	22.25 ± 0.63 ^fg^	20.75 ± 0.53 ^g^	69.42 ± 0.09 ^b^	28.11 ± 0.70 ^f^	99.55 ± 0.64 ^a^	48.70 ± 2.77 ^d^	62.13 ± 4.10 ^c^	99.47 ± 3.83 ^a^	41.17 ± 0.99 ^e^	**	**	**	6.44
Total FPA	1067.63 ± 1.66 ^f^	358.95 ± 3.08 ^i^	495.31 ± 3.63 ^h^	353.92 ± 0.98 ^i^	577.61 ± 0.98 ^g^	2121.55 ± 5.18 ^b^	1999.04 ± 7.07 ^c^	2956.06 ± 34.81 ^a^	1701.75 ± 5.10 ^d^	1219.39 ± 8.18 ^e^	**	**	**	22.91
BPA (µg g^−1^ dm)														
3.5-dihydroxybenzoic	52.37 ± 0.64 ^d^	23.11 ± 0.13 ^f^	24.83 ± 1.72 ^f^	54.66 ± 1.43 ^d^	31.08 ± 0.62 ^e^	81.97 ± 0.96 ^b^	25.05 ± 0.11 ^f^	156.81 ± 3.33 ^a^	60.54 ± 1.61 ^c^	85.42 ± 0.76 ^b^	**	**	**	4.57
3- hydroxybenzoic	-	-	-	-	-	196.55 ± 2.65 ^d^	217.51 ± 3.60 ^c^	466.56 ± 3.40 ^a^	191.62 ± 1.48 ^d^	450.32 ± 2.40 ^b^	**	**	**	6.26
Syringic	-	-	-	-	-	10.48 ± 0.50 ^c^	-	41.93 ± 0.96 ^a^	-	20.04 ± 2.09 ^b^	**	**	**	2.35
m-coumaric	-	-	-	-	-	27.70 ± 1.50 ^c^	16.59 ± 0.37 ^d^	61.73 ± 0.78 ^a^	-	35.48 ± 0.62 ^e^	**	**	**	1.84
Caffeic	-	-	-	-	-	12.57 ± 1.39 ^a^	-	-	-	-	**	**	**	1.39
p-coumaric	13.00 ± 0.62 ^f^	9.97 ± 0.20 ^fg^	6.26 ± 1.13 ^g^	21.70 ± 0.35 ^e^	19.96 ± 0.21 ^e^	31.51 ± 1.64 ^d^	38.56 ± 1.36 ^c^	54.48 ± 1.67 ^b^	30.65 ± 1.51 ^d^	65.49 ± 1.35 ^a^	**	**	**	3.63
Salicylic	29.81 ± 1.27 ^g^	28.20 ± 0.01 ^g^	44.01 ± 2.10 ^f^	42.85 ± 1.03 ^f^	25.07 ± 0.13 ^g^	133.07 ± 0.08 ^d^	250.37 ± 2.77 ^a^	83.08 ± 2.12 ^e^	141.03 ± 2.08 ^c^	217.50 ± 1.67 ^b^	**	**	**	5.12
Ferulic	18.87 ± 1.23 ^e^	12.36 ± 0.75 ^f^	10.49 ± 1.09 ^f^	26.26 ± 0.78 ^d^	18.43 ± 0.98 ^e^	33.58 ± 0.41 ^c^	29.65 ± 0.53 ^cd^	78.30 ± 0.42 ^a^	29.16 ± 1.40 ^cd^	44.94 ± 1.24 ^b^	**	**	**	4.77
Sinapic	91.84 ± 2.75 ^b^	63.06 ± 0.57 ^e^	40.95 ± 1.92 ^g^	111.67 ± 2.38 ^a^	72.04 ± 0.84 ^d^	86.98 ± 1.08 ^c^	54.29 ± 0.10 ^f^	60.50 ± 0.69 ^e^	51.98 ± 2.04 ^f^	64.46 ± 0.16 ^e^	**	**	**	4.86
Total BPA	205.89 ± 6.51 ^e^	136.70 ± 1.26 ^g^	126.53 ± 5.79 ^g^	257.14 ± 5.98 ^d^	166.58 ± 0.83 ^e^	624.07 ± 3.48 ^b^	632.02 ± 4.95 ^b^	1003.39 ± 12.19 ^a^	504.99 ± 3.21 ^c^	1000.48 ± 1.23 ^a^	**	**	**	15.33
TPA	1273.52 ± 4.85 ^e^	495.65 ± 1.92 ^h^	621.84 ± 2.16 ^g^	611.06 ± 6.96 ^g^	744.19 ± 2.50 ^f^	2745.62 ± 4.64 ^b^	2631.06 ± 2.83 ^c^	3959.45 ± 25.56 ^a^	2206.74 ± 17.03 ^d^	2219.88 ± 5.02 ^d^	**	**	**	32.81

**Table 3:** Contents of glucosinolate molecules (GS9: glucoarabin; GS10: glucocamelinin; GS11: homoglucocamelinin) and of total glucosinolates (GLSs) (mg g^−1^ dm) in seeds and sprouts of five of *Camelina sativa* (L.) Crantz cultivars (ALBA, CO46, CCE43, JOELLE, and VERA). Means of n = 2 replicates ± standard errors are depicted. *G*: genotype; *GS*: growing stage; *LSD*: least significant difference of interaction. Values in the same row followed by different lowercase letters are significantly different for *p* = 0.05. Significance of ANOVA: ** significant at *p* ≤ 0.01; * significant at *p* ≤ 0.05.

GLSs (mg g^−1^ dm)	Seeds					Sprouts					Significance			
	ALBA	CO46	CCE43	JOELLE	VERA	ALBA	CO46	CCE43	JOELLE	VERA	*G*	*GS*	*G × GS*	*LSD*_0.05_
GS9	3.00 ± 0.02 ^d^	1.45 ± 0.02 ^f^	0.84 ± 0.02 ^h^	1.40 ± 0.01 ^f^	1.20 ± 0.01 ^g^	6.12 ± 0.02 ^a^	3.68 ± 0.02 ^c^	2.98 ± 0.06 ^d^	3.91 ± 0.02 ^b^	2.37 ± 0.03 ^e^	**	**	**	0.080
GS10	2.51 ± 0.01 ^a^	1.16 ± 0.01 ^c^	1.10 ± 0.01 ^d^	1.34 ± 0.01 ^b^	1.10 ± 0.03 ^d^	0.22 ± 0.01 ^h^	0.31 ± 0.02 ^g^	0.55 ± 0.02 ^f^	0.91 ± 0.02 ^e^	0.26 ± 0.01 ^gh^	**	**	*	0.050
GS11	0.66 ± 0.01 ^a^	0.27 ± 0.01 ^g^	0.41 ± 0.01 ^d^	0.47 ± 0.01 ^b^	0.30 ± 0.01 ^g^	0.43 ± 0.01 ^c^	0.04 ± 0.01 ^h^	0.34 ± 0.01 ^f^	0.38 ± 0.02 ^e^	0.05 ± 0.01 ^h^	**	**	**	0.025
Total GLSs	6.16 ± 0.04 ^b^	2.88 ± 0.04 ^g^	2.34 ± 0.03 ^i^	3.21 ± 0.01 ^f^	2.59 ± 0.04 ^h^	6.77 ± 0.05 ^a^	4.02 ± 0.01 ^d^	3.87 ± 0.13 ^e^	5.19 ± 0.03 ^c^	2.68 ± 0.04 ^h^	**	**	**	0.110

**Table 4:** Linearity and homoscedasticity test for calibration plots, results for system-suitability study, detection, and quantitation limit values for glucosinolates (GLSs).

GLSs	Cc	R	Linear Equation	tR (Min)		A		N		T		LOD	LOQ
				Mean	RSD%	Mean	RSD%	Mean	RSD%	Mean	RSD%		
GS9	0.251	0.9984	y = 357.723 × x − 15.66	1.837	0.574	545.213	1.279	5396.532	1.148	1.298	1.356	0.025	0.083
GS10	0.230	0.9984	y = 357.723 × x − 15.66	2.969	0.313	483.704	1.389	13,668.939	1.014	1.448	1.335	0.052	0.174
GS11	0.250	0.9980	y = 357.723 × x − 15.66	4.302	0.224	1167.362	1.423	25,080.563	1.231	1.164	1.374	0.039	0.129

Mean of ten replications. GLSs: glucosinolates; GS9: glucoarabin; GS10: glucocamelinin; GS11: homoglucocamelinin; Cc: Cochran’s constant calculated; R: correlation coefficient; tR: retention time; A: peak area; RSD: relative standard deviation; N: number of theoretical plates; T: tailing factor; LOD: limit of detection; LOQ: limit of quantitation.

**Table 5:** Trueness (R′%) and precision (RSDr%) and Horwitz ratio value (HorRat) of the optimized method, based on three concentration levels (1, 5, and 15 mg g^−1^) of glucosinolate (GLS)-spiked *Camelina sativa* (L.) Crantz sprouts. N = 10. R’ = relative spike recovery; SD: standard deviation; RSDr = relative standard deviations of repeatability; HorRat = Horwitz ratio value. GS9: glucoarabin; GS10: glucocamelinin; GS11: homoglucocamelinin.

GLSs	Concentration Level (mg g^−1^)								
	1			5			15		
	R′%	RDSr%	HorRat	R′%	RDSr%	HorRat	R′%	RDSr%	HorRat
	(Mean ± SD)			(Mean ± SD)			(Mean ± SD)		
GS9	100 ± 2	1.8	0.2	102 ± 2	2.3	0.3	102 ± 3	2.5	0.4
GS10	92 ± 2	2.4	0.2	98 ± 2	2.4	0.2	102 ± 2	2.1	0.2
GS11	110 ± 2	1.4	0.3	102 ± 3	2.6	0.5	94 ± 2	2.2	0.4

## Data Availability

Not applicable.
